# Septoplasty: early (first year) and late (fourth year) post-operative results in 604 patients – ERRATUM

**DOI:** 10.1017/S0022215126104563

**Published:** 2026-07

**Authors:** R Haye, L K Døsen, M TarAngen, C Gay, M T Egeland, O Shiryaeva

Publisher Note: Readers, please note that an Accepted Manuscript version of this article was initially published twice in error. The duplicate had the following DOI assigned: https://doi.org/10.1017/S0022215121002966, which has since been redirected to this Version of Record.

This article was first published on 20th October 2021 which has been now updated from 26th November 2021.

## References

[ref1] Haye, R. et al. (2022) ‘Septoplasty: early (first year) and late (fourth year) post-operative results in 604 patients’, *The Journal of Laryngology & Otology*, 136(6), pp. 514–519. doi:10.1017/S0022215121003741. 34823616

